# Supply and demand of higher vocational education in China: Comprehensive evaluation and geographical representation from the perspective of educational equality

**DOI:** 10.1371/journal.pone.0293132

**Published:** 2023-10-19

**Authors:** Yong Han, Ruixing Ni, Yating Deng, Yuanyuan Zhu

**Affiliations:** 1 School of Geographic Sciences, Xinyang Normal University, Xinyang, 464000, China; 2 Key Laboratory of Geographic Process Analysis & Simulation Hubei Province, Central China Normal University, Wuhan, 430079, China; Chulalongkorn University, THAILAND

## Abstract

The imbalanced regional development of higher vocational education, particularly the disparity between the supply and demand of educational resources, has emerged as the primary factor impeding the provision of high-quality higher education in China during the establishment of a universal education system. Based on the 1,482 higher vocational education institutions recognized by the Ministry of Education of China in 2021 as the research objects, the development of higher vocational education in China was explored from the perspective of supply and demand using the entropy weight TOPSIS method and coupling coordination degree model. It was found that China’s higher vocational institutions were mainly located in provincial capitals, representing a point distribution pattern. From a comprehensive evaluation of the supply level, areas such as the Beijing-Tianjin-Hebei region, Yangtze River Delta, and central Henan Province have become the catchment areas for the development of higher vocational education, laying the foundation for regional network cooperation. From the perspective of educational equality, the higher vocational education in China was found to be sufficient to match the supply and demand, and a balance between supply and demand was apparent in provincial capitals. The coupling degree between supply and demand exhibited an “olive-type” spatial structure pattern, indicating that the development of higher vocational education in most cities in China is still in the transformation stage. The results provide a scientific basis for optimizing resources in the provision of higher vocational education.

## Introduction

The development of higher vocational education (HVE) has received increasing attention worldwide, particularly as countries seek to address skills shortages and prepare their workforce for the changing demands of the job market [1]. Production changes and increasing demand for decent work are also reflected in the increase in demand for and remuneration of higher skills [2], especially in times of global crises, such as the COVID-19 pandemic [3]. In this context, HVE is often designed to meet the needs of specific industries, meaning that graduates are well-positioned to enter fields where there is a high demand for skilled workers. This can lead to long-term career stability, as well as opportunities for advancement within the industry. The provision of decent work is an important aspect of the United Nations’ Sustainable Development Goals (SDGs). It is specifically addressed as SDG 8: “Promote sustained, inclusive, and sustainable economic growth, full and productive employment and decent work for all.” A higher level of vocational education can provide individuals with advanced skills and knowledge in their chosen field, leading to better job prospects and higher wages [4]. Moreover, HVE is an important component in promoting decent work because it provides individuals with the advanced skills and knowledge needed to succeed in the current competitive job market [5].

Many Asian countries, including China, are rapidly adopting new and emerging technologies in industry and agriculture. These new technologies generally need a higher knowledge-based system and enormous technical skills to exploit them efficiently. Many Asian countries, including China, Japan, and South Korea, have been investing heavily in vocational education and training as a means of ensuring that their workforce is adequately skilled and prepared for new industries and technologies [6,7]. In China, HVE refers to post-secondary education that offers vocational and technical training to prepare students for specific occupations or industries [8]. The Chinese government has been promoting the high-quality development of vocational education to meet the surging demand for the upgrading of industries and to support the country’s manufacturing base. The government has set a goal to establish a modern vocational education system in China by 2025, and to have China’s vocational education ranked among the best globally by 2035 [9]. Thus, the supply of HVE in China is increasing and is expected to continue to grow in the coming years. However, despite the progress made, there are still challenges presented by the development of HVE in China. One of the major challenges is regional inequality, which manifests in the uneven distribution of educational resources, variations in student satisfaction with the learning process, and differences in teacher-student interactions across regions [10–13]. These disparities have resulted in a shortage of skilled workers in some regions, despite the country’s large number of HVE colleges and graduates. In addition to the inter-regional differences in quantity and quality, China’s higher education development also faces a problem of structural imbalance. This structural imbalance and the insufficient educational provision has resulted in the supply of HVE being unable to meet the needs of individuals. By the end of 2022, China had 3,097,500 graduate students, 19,656,400 undergraduate students, and 16,709,000 vocational college students, accounting for 7.9%, 49.8%, and 42.3% of the total students, respectively, exhibiting an “olive-type” structure with a large middle and two small ends, which was very different from the “pyramid” structure of international higher education. These structural problems have the spatial appearance of regional differences, and can be attributed to the mismatch between supply and demand, thus leading to the inequality of higher education provision. It is important to answer the following questions. How can policymakers address the issue of underutilized or overburdened HVE institutions in certain regions? How can businesses and industries located in underdeveloped regions be encouraged to invest in training programs for local workers? What factors contribute to the uneven distribution of HVE institutions across different regions in China? These questions highlight some of the key challenges facing policymakers and educators as they work to promote greater equity and access within China’s HVE system, particularly when addressing the regional imbalances between supply and demand.

To achieve this goal, our work had two main objectives. First, we developed a comprehensive evaluation index system to assess the supply of HVE and reflect the practical requirements needed for its high-quality development. Second, we used geographical techniques to map the regional differences in HVE supply and demand, as identify the factors that influence these differences. For the first time, this study accurately described the spatial distribution characteristics of HVE institutions in China, which further complemented the existing spatial heterogeneity characteristics of HVE at the research scale.

The remainder of the paper is organized as follows. A brief literature review is provided in the second section. Building on the previous literature, our research methods, including the methodology applied and data used for the analysis, are introduced in the third section. The results of the analysis are presented in the fourth section, and the fifth and sixth sections are the discussion and conclusions, respectively.

## Literature review

### Regional educational equality and HVE

The geography of education provision has become an important issue in the 21^st^ century. With the increasing political attention given to education, research within the field of the geography of education provision has also increased [14,15]. Generally, the geography of higher education explores questions such as: where are higher education institutions located, and why [16–18]? How do different regions, countries, and cultures approach higher education [19]? What factors influence the growth and development of higher education systems, and how do they change over time [20]? Higher vocational education is an important part of the higher education system. The goal of HVE is to provide individuals with the vocational skills, knowledge, and abilities necessary for qualified vocational activities, and to acquire necessary vocational experience through a standardized educational process in response to the changing labor environment. The development of HVE is a significant concern for all countries, and the regional inequality of HVE supply and demand is also a major issue worldwide [21]. The development of HVE varies widely across different regions and countries, and this spatial variation has become an important dimension of education equality. The spatial dimension of HVE is an important issue in its sustainable development. One possible reason for the development of spatial variations in HVE is that certain vocational programs, such as those in the fields of agriculture, forestry, or environmental science, may require knowledge of local geography to understand regional variations in climate, soil types, and natural resources. Additionally, some vocational programs may have a strong focus on local industries or businesses. Therefore, each vocation has its own spatial distribution pattern [22]. In these cases, understanding the geographical context of an area can be important for students to develop skills that are relevant to their future careers. Vocational decisions, particularly for young people growing up in challenged localities, are therefore spatial decisions. Some program choices lead to locally provided schooling, which may tie graduates into the local labor market for life [22]. Furthermore, studying geography can also help students to develop critical thinking skills and gain a broader perspective of global issues. This can be beneficial for individuals pursuing any type of higher education program.

Although there are various definitions of HVE, which encompass the different types of institutions governed by different arrangements in various locations, they can be viewed as an equity strategy to widen access to higher education, especially for those traditionally excluded by academic education programs. In relation to social justice and equity, social inequalities are considered to be exacerbated for those deemed underqualified for work in knowledge-driven economies. New opportunities are therefore needed to widen participation in higher education. Consequently, in countries where the expansion of HVE qualifications takes place in institutions that have distinctive traditions of serving their local communities and employers, the expansion of higher vocational qualifications is viewed as an equity strategy to widen access to higher education **[23]**. Increasing the number of higher education providers or the range of higher education-level offerings and thereby changing the institutional boundaries and remits of post-school educational providers has been the most widely adopted approach. Universal systems of higher education have subsequently been created in many countries, and a system change through expansion is one of the main approaches countries have adopted to widen access to higher education for those who traditionally have not participated **[24]**. With the expansion of higher education, more people from all backgrounds have been able to access higher education; however, inequalities between social groups tend to continue. Individuals from underrepresented and marginalized backgrounds find access to lower status forms of higher education is made easier than access to the more selective and elite forms **[25]**, which ultimately aggravates educational inequity. In systems such as that of Australia that is formally status averse, at least with respect to differences between universities, since 1973 the policy approach to equity has been to extend higher education to under-represented social groups at the margins of participation rather than redistributing social access to elite universities **[26]**. Therefore, HVE is considered to provide a connection with higher education systems and to expand opportunities to higher education qualifications for those who have not traditionally participated at this level.

### The sustainable development of HVE from the perspective of the regional balance between supply and demand

Vocational education and training (VET) has a prominent place in the 2030 Agenda for Sustainable Development. Equal access to affordable, high quality VET is a target of the SDGs, together with a commitment to substantially increase the number of youths and adults with the relevant skills for employment, decent jobs, and entrepreneurships by 2030. Vocational education and training programs will be key to achieving both the 4^th^ SDG for quality education and the 8^th^ SDG for good jobs and economic growth [27]. The sustainable development of HVE from the perspective of the regional balance between supply and demand should be considered from two aspects. First, the vocational education institutes and programs offered in a particular region are aligned with the needs of the local industries and labor market. The vocational education and training market experienced significant growth among the public during 2021 in the world. This trend is expected to continue, with even higher growth rates anticipated from 2022 to 2028. The need for skilled workers in various industries drives the increasing demand for vocational education and training [28,29]. However, a pre-requisite for the positive influence of VET on economic development is the appropriate design of relevant curricula based on a market needs identification and analysis [30]. For example, in England, the allocation of HVE resources has shifted away from Keynesian government regulations towards apprenticeship programs, following changes in the national leadership of HVE establishments [31,32]. In the United States, there is a focus on developing basic skills and abilities through HVE to help individuals navigate economic crises. Thus, HVE resources are allocated based on both market demand and government regulation, with state governments approving vocational colleges based on regional economic development targets, leading to a differentiated distribution within each state [33,34].

The second important aspect of HVE is its role in promoting social sustainability by increasing access to higher education for students who are unable to enter traditional academic education programs, which is a form of educational equity. By ensuring that HVE is accessible, equity can be promoted for students who are unable to access a traditional academic education. This will help to reduce the gap in opportunities and outcomes between students from different backgrounds, and promote a more equitable society. The expansion of the higher education system has been shown to increase participation rates [35], which can be understood as creating greater equity through increasing availability. However, the precondition for increased participation is that HVE has a lower barrier to accessibility than higher academic education, which is the reality in most countries. We therefore believe that under the background of higher education expansion, a relative equity will develop between regions in China in terms of access to HVE opportunities.

In summary, most recent major studies of higher education have focused almost exclusively on university education. A dominance of research on universities rather than on alternative providers offering HVE in expanded systems was also identified in a recent systematic review **[36]**. The present study of the geography of higher education considered the geographical methods used to explain the development of HVE, which have led to regional differences in educational inequity not being effectively described. The few studies that have considered the regional differences of HVE provision have used official statistics at the provincial scale **[37]**. These data accurately describe the regional heterogeneity characteristics, especially for countries like China with its massive territory. Supply and demand in the context of higher education can be quite difficult to define and definitions may vary. Previous studies of higher education have made some suggestions for the measurement of supply and demand. Rothschild and White acknowledge that a university’s “production level” or supply is “the number of students to admit” or enrollment spaces available **[38]**. To broadly define the supply of higher education, the “production levels” at each university must be aggregated and the number of universities available must be taken into account **[39]**. Finally, the demand level of HVE mainly considers the need for enterprise and markets, but does not fully consider the needs of students. In a large country such as China, many school-age people directly flow to the labor market every year because they do not enter HVE, which creates unfavorable conditions for future industrial transformation and development.

## Materials and methods

### Index system

Vocational education has different characteristics from ordinary education programs with a focus on the two elements of a specific occupation and education. If the promotion of economic development and decent employment is regarded as the utilitarian goal of vocational education from an economic function perspective, then the promotion of personal development and social equity is the human nature goal from an educational perspective [40]. Therefore, a balance between the supply and demand of vocational education is required to highlight the educational opportunities of social equity, with the recognition that educational products carry social services.

The supply of vocational education therefore refers to the educational opportunities provided to individuals and the educational products provided to society. Educational opportunities occur at the starting point of the vocational education process, which is reflected in the quantity and quality of educational institutions; whereas educational products occur at the end of the vocational education process, which is reflected in the quantity and quality of graduates of educational institutions. The selection of indicators in the supply dimension was made with reference to our previous research [16]. The demand for vocational education is determined by the needs of society and individuals to pay for vocational education products and vocational education opportunities, i.e., the need to develop social functionality and utility, which occurs during the process of vocational education. Here, the demand for vocational education products is a function of the demand for vocational education opportunities. Demand for vocational education products should meet the needs of social development. Vocational education opportunities designed to meet the needs of personal development are ensured in both the temporal and spatial dimensions. Table 1 shows the indicators used for the evaluation of the supply and demand of HVE. Table 1 shows the indicators used for the evaluation of the supply and demand of HVE.

**Table 1:** The evaluation of the supply and demand of HVE in China.

First indicator	Second indicator	Third indicator	Indicator instructions
Supply	Opportunity	Number of students enrolled (X_1_)	Annual number of higher education enrollments in the region
		Number of teachers (X_2_)	The number of teachers in higher education in the region
		Number of universities (X_3_)	The number of colleges and universities in the region
	Process	Skills cultivation (X_4_)	The ability of colleges and universities to train students
		Teacher performance (X_5_)	Performance level of teachers in colleges or universities
	Outcome	Employment quality (X_6_)	The quality of employment for graduates
		Enrollment rate (X_7_)	Proportion of postgraduates continuing education
Demand	Student group	Priority of selection (X_8_)	The examination pass rate used by students to prioritize their university selection
		Student demand (X_9_)	Number of high school graduates
	Social subjects	Social population demand (X_10_)	Proportional demand for vocational education among the school-age population
		Social employment demand (X_11_)	The proportion of the employed population with college degrees

### Main methods

#### Entropy weight TOPSIS

The entropy weight TOPSIS method is essentially an improvement of the traditional TOPSIS evaluation method. The weight of the evaluation index is determined through the entropy weight method, and then the TOPSIS method uses technology to approximate the ideal solution. The entropy weight method is used to objectively determine the weight according to the information provided by each evaluation index. The entropy weight number can objectively reflect the importance of an index in the index system when making decisions [41]. The calculation steps are as follows:

The dimensionless matrix is obtained by the normalization method:

$$Y_{ij}=({y_{ij})}_{m*n},y_{ij}=\frac{x_{ij}}{\sum_{i=1}^{m}x_{ij}}$$ (1)

On this basis, the weighted normalized decision matrix is calculated:

$$P={\left(p_{ij}\right)}_{m*n}$$ (2)

The positive and negative ideal solutions of the matrix are determined:

$$P_{j}^{+}=max(p_{1j},p_{2j},\cdots ,p_{mj})$$

$$P_{j}^{-}=min(p_{1j},p_{2j},\cdots ,p_{mj})$$ (3)

The distances between the index vector of each evaluation object and the positive and negative ideal solutions are calculated:

$$d_{i}^{+}=\sqrt{\sum_{j=1}^{n}{\left(p_{ij}-p_{j}^{+}\right)}^{2}},d_{i}^{-}=\sqrt{\sum_{j=1}^{n}{\left(p_{ij}-p_{j}^{-}\right)}^{2}},(0\leq d_{i}^{+},d_{i}^{-}\leq 1)$$ (4)

The degree of closeness between each evaluated object and the optimal value is determined:

$$C_{i}=\frac{d_{i}^{-}}{d_{i}^{+}+d_{i}^{-}},(0\leq C_{i}\leq 1)$$ (5)

where *i* represents the city; *j* represents the index; *m* is the number of cities; *n* is the number of relevant indicators to evaluate vocational education; $P_{j}^{+}$ is the positive ideal solution vector for each index and $P_{j}^{-}$ is the negative ideal solution vector for each index; $d_{i}^{+}$ and $d_{i}^{-}$ are the euclidean distances from each city to the vector of positive and negative ideal solution, respectively; and *C*_*i*_ is the final result following a comprehensive evaluation of vocational education in each city.

### The coupling coordination degree model (CCDM)

Based on the concept of capacitive coupling and its coupling coefficient model in physics, we built a CCDM to evaluate the coupling relationship between multiple systems or factors [42]. The CCDM can be expressed as follows:

The degree of coupling is determined:

$$C={\left\{\frac{\left(f(U)*g(E)\right)}{{\left(\left[f(U)+g(E)/2\right)\right]}^{2}}\right\}}^{1/2}$$ (6)

The overall effect level is calculated:

T=αf(U)+βg(E) (7)

The degree of coupling coordination is calculated:

$$D=\sqrt{C*T}$$ (8)

where C represents the degree of coupling; *f*(*U*) is the supply of vocational education; and *g*(*E*) is the demand of vocational education. Here, *f*(*U*) and *g*(*E*) were both calculated by *Si* as described above. *D* is the degree of coupling coordination and *T* reflects the overall effect level between supply and demand in vocational education. *α* and *β* represent the contributions of supply and demand, respectively.

Combined with previous research, the results are shown in Table 2.

**Table 2:** Classification of the CCDM.

First Grade Index	Second Grade Index	Features
Balanced development (0.8 < D ≤ 1.0)	Quality coordination (0.9−1.0]	The supply and demand of HVE are highly balanced, i.e., they are fully matched in quantity and highly-developed in quality. The prioritization of educational resource investment takes precedence over the expansion of education scale, with HVE institutions enrolling a larger number of students than undergraduate colleges and universities. Through HVE training, students can gain access to more educational opportunities, better quality education, and improved prospects for decent job opportunities.
	Good coordination (0.8−0.9]	
Transitional Development (0.5 < D ≤ 0.8)	Intermediate coordination (0.7−0.8]	The supply of HVE institutions barely meets local students and social needs in quantity, and the quality of HVE needs to be improved. The provision of educational resources should align with the expansion of educational scale, and the enrollment scale of HVE should be in line with that of undergraduate universities. Individual students should have access to sufficient educational opportunities and employment prospects through HVE training.
	Primary coordination (0.6−0.7]	
	Forced coordination (0.5−0.6]	
Unbalanced Development (0.0 < D ≤ 0.5)	Borderline disorder (0.4−0.5]	The supply of HVE institutions cannot meet the needs of local students and society, with a serious mismatch in quantity and quality. The investment in educational resources lags behind the expansion of the education scale, and the enrollment scale of HVE is lower than undergraduate universities. Individual students are unable to access sufficient educational opportunities, resulting in a subpar quality of education. Consequently, individual students perceive a lack of future prospects through HVE training.
	Mild disorder (0.3−0.4]	
	Moderate disorder (0.2−0.3]	
	Severe disorder (0.1−0.2]	
	Extreme disorder (0−0.1]	

### Data resources and processing

**Fig 1:**
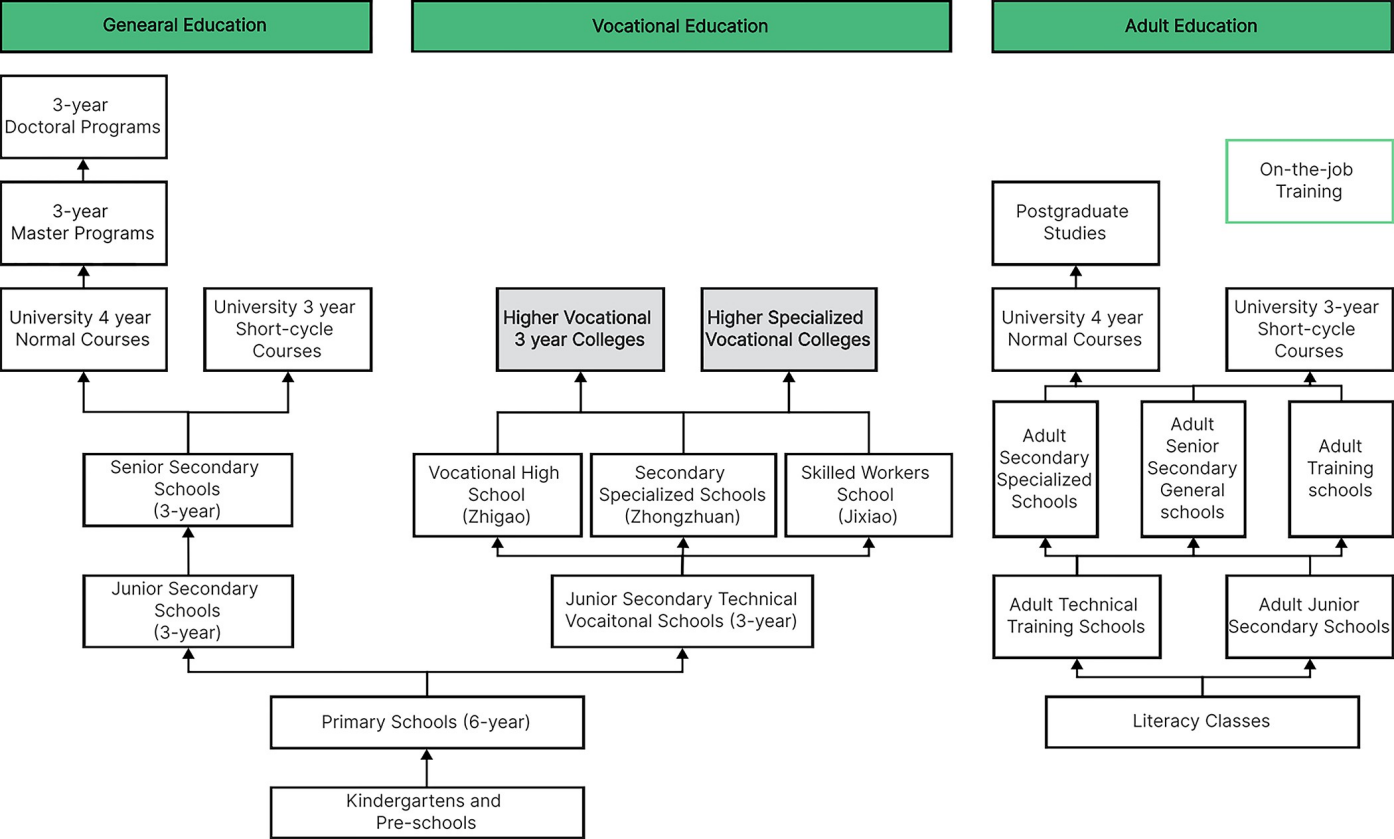
The education system in China and the research objects (in the gray box) in the study.

The study included all the universities on the Chinese mainland, excluding Hong Kong, Macao, and Taiwan. Chinese mainland, Hong Kong, Macao, and Taiwan have different education and management systems with different statistical standards, and the Ministry of Education does not provide data for these regions. The 1,482 HVE institutions listed by the Ministry of Education in 2021 were taken as the objects of our study (Fig 1), and the data were averaged to municipal units. A spatial statistical analysis was conducted for the municipal spatial units. The data were obtained from two sources. The first was data on educational opportunities (the X_1,_ X_2,_ X_3,_ X_9,_ X_10,_ and X_11_indicators) in the 2020 China Statistical Yearbook, The China 2020 Urban Yearbook, and The Statistical Bulletin of Urban National Economic and Social Development in 2020. The second was data on educational processes and \outcomes (the X_4,_ X_5,_ X_6,_ X_7,_and X_8_ indicators) in Choosing a University and Select a Major in 2020 (Vocation Universities edition). This is a reference book for college entrance examination applicants with university rankings, subject category rankings, and major rankings, supplemented by teacher level rankings and graduate quality rankings. The book is produced by the China Statistics Press and Professor Wu Shulian. It has been published continuously for 20 years and has a high degree of influence on the selection of schools in China’s college entrance examination.

The rank correlation coefficient was used to assess the relationships among the data. The X_1_, X_2_, and X_3_ variables were statistical data, whereas the other indicators were rank data. To maintain consistency, the statistical data were graded for processing. The specific methods used were as follows. The overall data were divided into 11 grades from small to large, and the larger the grade score, the higher the original data level. Grades 1 and 2 accounted for 15% of the overall data; grades 3 to 8 accounted for 10%; grade 9 accounted for 5%; grade 10 accounted for 3%; and grade 11 accounted for 2%. Taking Nanyang Medical College for example, its raw data showed the number of teachers was 3000, the priority of selection was A++, the quality of admission was A++, the value of skill cultivation was 28.25, the performance of teachers was A++, the value of scientific research 6.97, the teachers performance level A++, the quality of employment was A++, and the enrollment rate was A++. After reclassification, the indicator with the highest grade was ranked eleventh, and was given the value of 11, and so on. The indicators above were then reclassified and revalued as 11 grades (indicators of the number of teachers, the selection priority, quality of admission level, teachers’ performance, etc.).

## Empirical results

### Spatial distribution of HVE institutes in China

**Fig 2:**
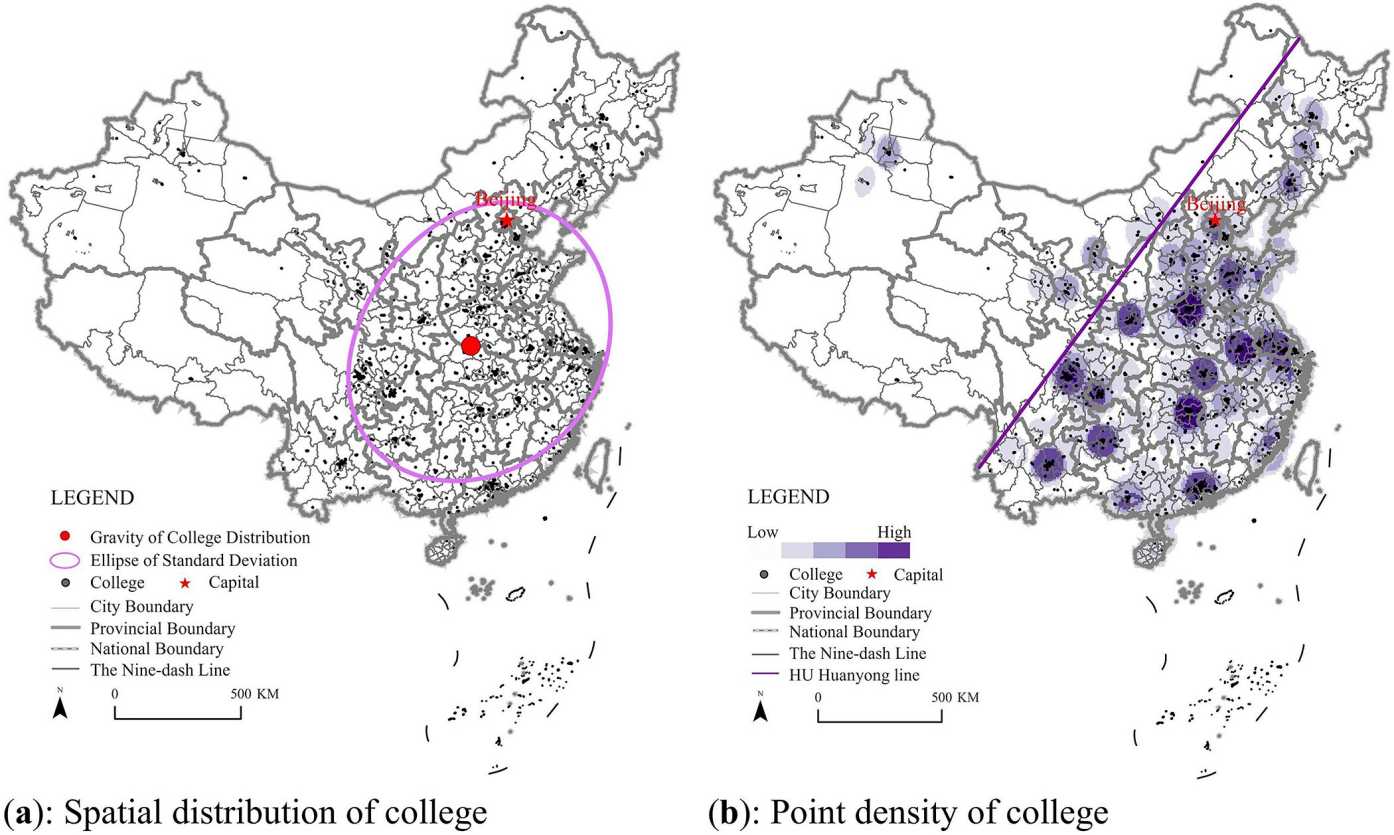
The spatial distribution characteristics of HVE colleges.

To evaluate the spatial distribution characteristics of HVE institutes in China, the ArcGIS Pro software was used to analyze HVE’s gravity, standard deviational ellipse, and point density estimation. The results showed that the spatial distribution center of Chinese HVE institutes was located in Xiangyang City, Hubei Province, with the institutes mainly distributed in the “cross-type regions,” which are the regions from Chengdu City to Shanghai City as the horizontal axis and from Beijing City to Guangzhou City as the vertical axis. The analysis of the standard deviational ellipse revealed that the gap between the long and short axes of the ellipse had a distance of 2.54 map units, and the deflection rate was 60.50 degrees, clearly indicating that HVE institutions in China were distributed from northeast to southwest. At the same time, the elliptical circumference had a length of 60.50 map units and an area of 283.69 map units, indicating that the distribution range of Chinese HVE institutions was relatively wide, and the distribution direction was consistent with the Heihe-Tengchong Line in China (Fig 2A). From the perspective of China’s four major regions, the eastern region of China is home to 522 HVE institutes, while the central region boasts 423, the western region has 414, and the northeast has 123. Among them, the eastern region accounted for 66% of the top 100 institutes in terms of comprehensive strength among vocational colleges, while the central region accounted for 19%, and the western region accounted for 15%. Notably, no one from the Northeast region made it to the top 100. In terms of the top 1000 ranking, the eastern region constituted 39.3%, followed by the central region with a share of 28%, then the western region with a proportion of 25.6%. The Northeastern part contributed only to a minor extent at 7.1%. According to the point density analysis, HVE institutions in China were mainly distributed in the central, eastern, and southern regions, with a few in the western and northern regions, and the high density points were mainly concentrated in provincial capitals and municipalities directly under the Central Government. (Fig 2B).

### Regional inequality in the supply and demand of HVE in China

#### The geographic representation of HVE supply

**Fig 3:**
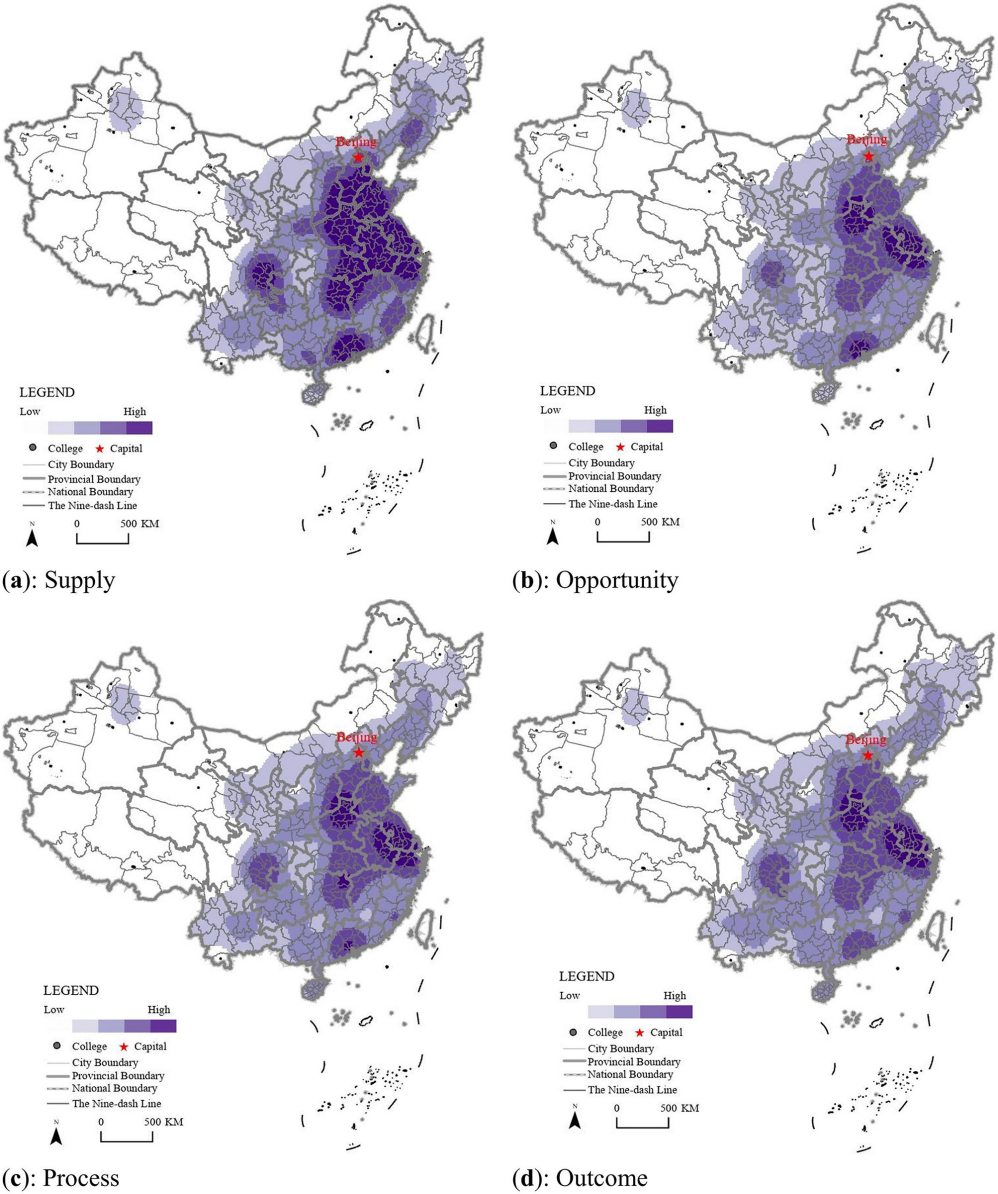
The geographic representation of HVE from the supply dimension in China.

To evaluate the degree of regional inequality of HVE supply in China, the entropy weight TOPSIS method and a kernel density estimation were used to describe the spatial pattern. The estimated values were visually represented using the natural fault point classification method based on ARCGIS Pro, with a search radius set to 0.8 and the extent of processing aligned with the geographical boundaries of China. The results indicated that there was a gradient difference law in the degree of supply from east to west. The results were visually presented using the natural fault point classification method based on ARCGIS Pro and Fig 1 displays the spatial distribution of supply in China. This study provides a multi-dimensional perspective on the evaluation of supply and highlights the need for new data sources and geographical statistical methods to further research the issue of regional inequality in the HVE supply in China (Fig 3A).

First, from the perspective of the educational opportunity, the supply of HVE in China presented a multi-core distribution pattern. The central and eastern regions had the greatest access to vocational education institutes with the most prominent regions being Henan Province, Yangtze River Delta, and Pearl River Delta. The northwest region had far lower levels of access than in the central and eastern regions, and was also lower than in the southwest region (Fig 3B).

Second, the two dimensions of process and outcome were the direct embodiment of educational opportunity. The ability to reflect the educational process of skills cultivation, scientific research, and serving society were directly related to the national, provincial, and municipal local financial allocation, and HVE institutes were therefore located close to the economically developed areas or close to the provincial capital city for resource access convenience. Skills cultivation was directly related to the quantity of students. Therefore, the three dimensions of educational process, outcomes, and inputs displayed similar spatial pattern characteristics (Fig 3C and 3D), with only slight differences in their representation of the energy center and range of influence. The supply of HVE was still dominated by opportunity in central and eastern China, with educational access having the characteristics of geographical proximity, and the educational process and outcomes were dependent on the input dimension.

#### The geographic representation of HVE demand

**Fig 4:**
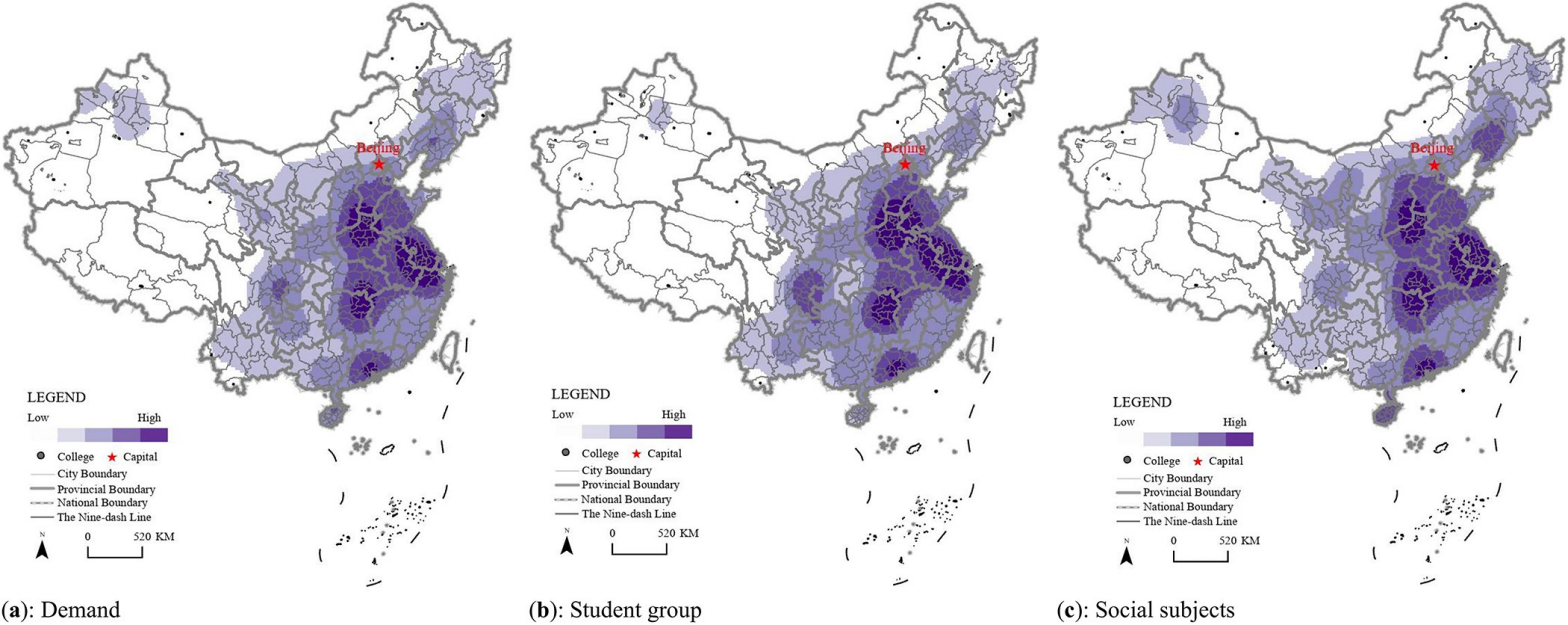
The geographic representation of HVE from the demand dimension in China.

The entropy weight TOPSIS method was used to calculate the indicators and estimate their values visually using the natural fault point classification method through ARCGIS Pro. The HVE demand presented a geographical pattern with high-values in the east and south, and low-values in the west and north. The high-value regions were mainly concentrated in Henan Province, Jiangsu Province, Hunan Province, Pearl River Delta, and Yangtze River Delta from the demand dimension (Fig 4A).

The higher-value regions in the HVE demand in China from student groups and social subjects were mainly located in the central region of China, the Yangtze River Delta region, and the Pearl River Delta region, especially in Henan, Hunan, Jiangsu, and Guangdong provinces (Fig 4B and 4C). This spatial pattern explicitly expressed the principle of proximity to HMV entrance opportunities in China. Taking 2021 as an example, the proportion of students within each province enrolled at HMV institutions ranged from 28% to 89%, including Beijing (89.60%) and Shanghai (72.23%) in east China, which was also consistent with the spatial pattern from the statistical data. However, from the perspective of student quality, excellent students still tended to enter HVE in the eastern provinces such as Shanghai, Hangzhou, and Suzhou. For example, students originating from other provinces accounted for 67% of all students in Shanghai in 2021. Therefore, there was a problem due to the large number of students taking the *Gaokao* (college entrance examination), but a low undergraduate admission rate, leading to a large gap in demand for further education. Taking 2021 as an example, the number of students taking the *Gaokao* was 104 million in Henan Province, 79 million in Shandong Province, and 78 million in Guangdong Province, ranking first, second, and third in China, respectively. The admission rates for higher academic education in these provinces were 41.5%, 43.08%, 51.28%, and for HVE were 23.45%, 35.24%, 24.46%, respectively. The demand gaps in the three provinces were approximately 375,000, 173,000, and 195,000, respectively.

#### The coupling coordination degree of both supply and demand

**Fig 5:**
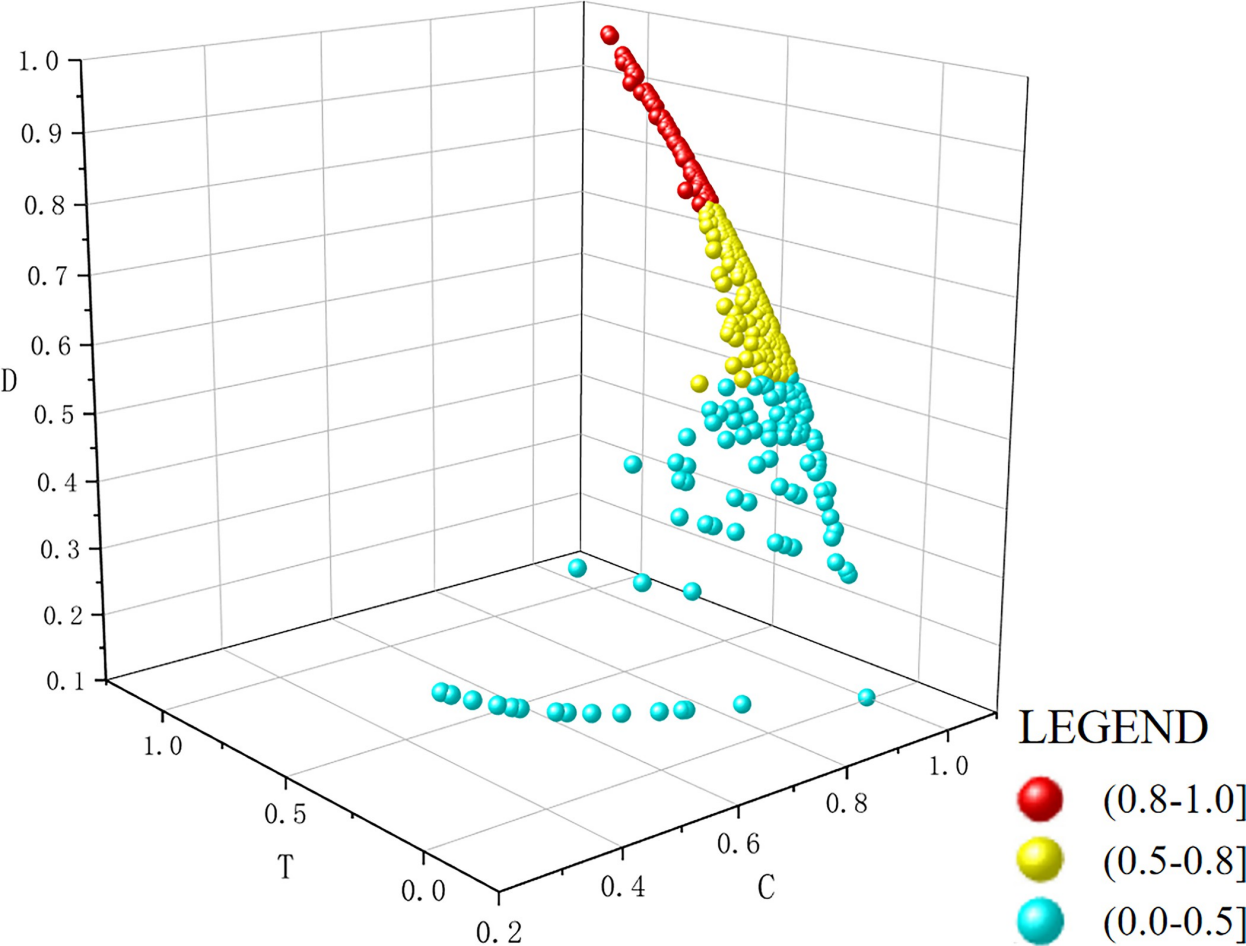
The coupling coefficient between supply and demand in each city in China.

Using the coupling coordination model to calculate all indicators of supply and demand, the results were divided into a coordination value T, coupling value C, and coupling coordination value D. The values of the three parameters were visualized in a 3D scatter plot using Origin software (Fig 5). The results showed that, in 56 cities the D-values ranged from 0.8 to 1.0, accounting for 15.22% of all cities in China, most of which were provincial capitals. In detail, 18 (56.25%) of the provincial capitals (there are 32 provincial-level administrative regions in China, excluding Hong Kong, Macao and Taiwan) were in the stage of quality coordination, and the remaining 14 (43.75%) provincial capitals were in the stage of good coordination. The number of cities in which the D-values ranged from 0.5 to 0.8 was 211, accounting for 57.34% of all cities in China, indicating that the development of HVE in these cites was in a transitional stage. The number of cities in which the D-values ranged from 0.0 to 0.5 was 101, accounting for 27.44% of all cities in China, indicating that the development of HVE in these cites was in an unbalanced stage.

**Fig 6:**
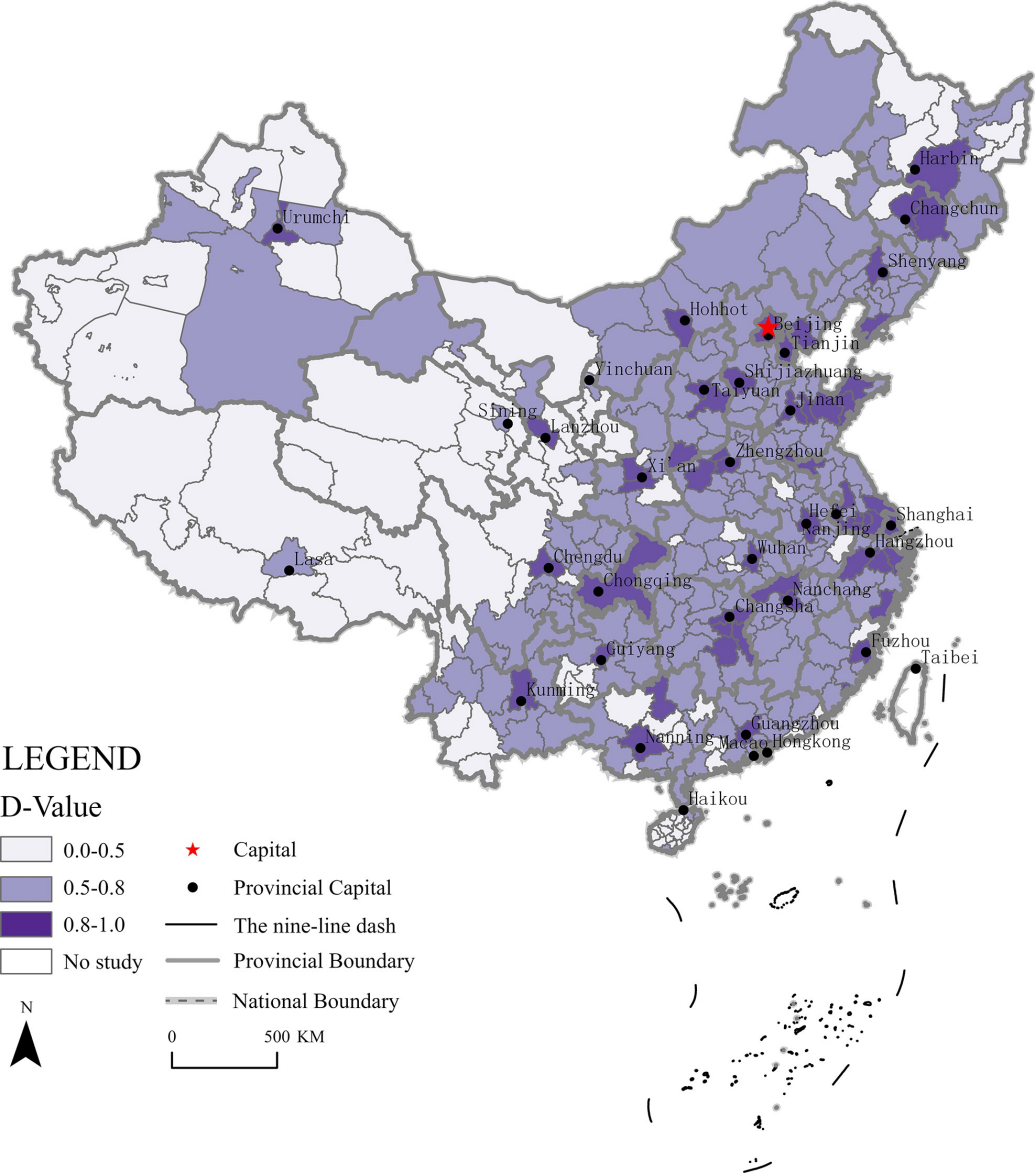
The geographic representation of the coupling coefficient between supply and demand in each city in China.

There was a large regional heterogeneity in the coupling and coordination of supply and demand of HVE in China. There were more cities with a high degree of coupling and coordination in the east than in the west, and they were mostly located in coastal areas. The areas with a high degree of coupling and coordination were mostly located in central regions and were largely provincial capitals and their surrounding areas. The areas with a high degree of coupling and coordination in the west were located exclusively in provincial capital cities (Fig 6). The reasons behind this geographical pattern were as follows. First, there was a highly coupled relationship among social subjects. The economic development of these regions has been largely dependent on enterprises, and their employment standards require training at the junior college level or above. A high demand for vocational education has arisen among the local population in an attempt to acquire better quality employment. Second, the supply and demand of students are highly coupled and coordinated. The area has a large permanent resident population and is one of the most densely populated areas in China, with a large population base and a large number of students.

## Discussion

China, like other developed counties, has undergone an expansion in its higher education provision from the mass stage to universal stage according to Trow’s model, which is a conceptual framework proposed by Clark Kerr and Martin Trow to explain the different stages of higher education development in different societies. Academic education, rather than vocational education, was responsible for most of the expansion, which has resulted in an over capacity in higher education **[43]**, especially in academic education. It is therefore expected that vocational education will play a more significant role in future higher education in China.

This study focused on the supply and demand balance of China’s HVE provision from the perspective of education equality, which has enhanced the demand dimension of HVE. The development of higher education in China is still in the stage of scale expansion. The ultimate goal of higher education is to ensure the entry of a high-quality labor force into the labor market. However, despite notable academic education achievements among students there are still structural unemployment issues, and the accessibility of HVE is an important way to ensure educational equity. The spatial pattern of HVE, in both the supply and demand dimensions, showed a concentration in the provincial capitals in China. Under the conditions of constant individual demand, only in provincial capitals could the individual and social needs for the supply of higher education resources be met. This was also apparent from the fact that the establishment of HVE in China had the characteristics of a near domain with the province as the unit of resource allocation. Additionally, the supply and demand relationship of HVE in China displayed an “olive-type” spatial structure pattern, indicating that the development of HEV in most Chinese cities is in the transitional stage. The olive model of spatial structure is relatively stable, which is the prerequisite for realizing the equity of HVE in society as a whole.

Our study accurately described the spatial distribution characteristics of HVE institutions in China. At the research scale, it further complemented the existing spatial heterogeneity characteristics of HVE provision in China. The application of geographic methods has filled the middle and micro scale research gap in the study of HEV in other disciplines. Previous studies only explored the spatial agglomeration issues of HVE at the quantitative level. Our study considered both the spatial distribution characteristics of individual HVE institutions and the development quality of HVE. It provides a detailed description of the spatial structural features of China’s HVE. Previous studies of higher academic or vocational education development have mainly focused on a single dimension in the external or internal systems of education. However, the transition of China’s HVE to high-quality oriented development requires a shift towards a multidimensional evaluation of HVE. Furthermore, the contemporary trend in higher education systems is a move towards universal access, with some institutions already on the verge of achieving this goal. The global experience in higher education development has shown a move toward diversity and stratification during the universal stage [44]. In this study, CCDM models were applied to identify the different stages of HVE development following the universal stage in China. First, in the stage of opportunity equalization (0.0 < D ≤ 0.5), a crucial objective is to attain parity in higher education opportunities. This entails not only ensuring universal access to higher education but also guaranteeing an equitable distribution of opportunities among students from diverse backgrounds, encompassing both urban and rural areas as well as affluent and underprivileged families. Second, in the stage of education quality improvement (0.5 < D ≤ 0.8) the emphasis is placed on elevating the standard and caliber of education to ensure that universal higher education fosters an optimal teaching and learning environment, while equipping graduates with proficient knowledge and practical skills. Third, in the stage of adapting to employment demand (0.8 < D ≤ 1.0) higher education institutions should prioritize the cultivation of skills that align with the evolving job market demands and equip students with career-oriented skills and knowledge to facilitate their seamless adaptation to dynamic changes in employment opportunities.

Finally, the issue of the balanced development of HVE was explored from a geographic perspective. The ultimate goal was to address the problem of sustainable HVE development between regions in China. With the gradual disappearance of China’s demographic dividend and the upgrading of industries, the cross-regional mobility of labor required by the manufacturing industry has gradually weakened, leading to increased local agglomeration. At the same time, low-skilled labor is increasingly lacking in market competitiveness, and vocational education and training have become an effective means of promoting China’s sustainable economic development. However, the non-equilibrium characteristics of the supply-demand relationship in China’s HVE between regions are unfavorable for achieving regional coordination and sustainable development. Based on the results of this study, there is a need to provide services for the planning of high-quality development and resource optimization of China’s HVE. For example, localities with supply-demand imbalances should increase their investment in vocational education resources, achieve cross-regional cooperation and collaboration, and comprehensively develop various vocational education institutions that are coordinated with the regional and surrounding industries. The reliance on urban agglomerations with HVE college resources can drive the development of lower-quality resources in surrounding counties that are connected to related professions, and continuously improve their social recognition. Catchment areas with a balanced supply and demand should leverage their comparative advantages within the region, strengthen the driving force and coordination of urban agglomerations, and enable the positive effects of the spatial spillover of vocational education policies to play a positive role, thereby optimizing the HVE resources in regions with a poor supply and demand balance.

## Conclusion

The development of China’s HVE and its supply-demand relationship still exhibits regional imbalances. Our analysis revealed several key characteristics of this development pattern. First, from the perspective of supply and demand, we observed three stages in the development of HVE after the popularization of higher education in China: the stages of opportunity equalization, education quality improvement, and adapting to employment demand. Second, the supply and demand relationship of HVE in China exhibited an “olive-type” spatial structure pattern, with D-values ranging from 0.8 to 1.0 accounting for 15.22% of all cities in China, D values of 0.5 to 0.8 accounting for a further 57.34%, and D values below 0.5 accounting for the remaining 27.44%. Third, HVE institutions were predominantly concentrated in provincial capitals and municipalities, and therefore exhibited a high degree of coupling, coordination, and alignment with the spatial distribution of economically developed regions. We suggest that this non-equilibrium spatial pattern arises from the inherent structural conflict between supply and demand in the development of HVE. However, the insufficient supply of HVE was largely due to several structural contradictions. First, Chinese students and parents have long favored academic education, resulting in many high school graduates choosing to study academic programs rather than choose vocational education. Second, the problem of a mismatch between the setting of majors in HVE and market demand has caused significant differences in educational outcomes, which has generated a psychological expectation that HVE is an insufficient approach for educational development in most families. The newly revised China Vocational Education Law in 2022 focuses on ensuring that vocational education has the same important status as general education, and aims to achieve the connection and integration of vocational and general education. In the future, China should optimize the allocation of hierarchical and classified vocational education resources based on local and surrounding economic development, industry, and skills demand, and with regard to the existing differences in the spatial distribution of vocational education resources. This policy demand is also an academic issue that requires further attention in the future.

## Supporting information

S1 Fig The geographic representation of HVE from the supply dimension in China. (XLSX)

S2 Fig The geographic representation of HVE from the demand dimension in China. (XLSX)
